# Contrasting Seasonal Distribution Patterns of Two Boreal Aerial Hawking Bat Species in Finland

**DOI:** 10.1002/ece3.70599

**Published:** 2025-01-16

**Authors:** Piia Lundberg, Miika Kotila, Katarina Meramo, Kati M. Suominen, Miina Suutari, Tia‐Marie Pietikäinen, Ville Vasko, Thomas M. Lilley

**Affiliations:** ^1^ Finnish Museum of Natural History University of Helsinki Helsinki Finland; ^2^ Helsinki Institute of Sustainability Science (HELSUS) Department of Forest Sciences, University of Helsinki Finland; ^3^ Department of Biology University of Turku Turku Finland; ^4^ Water and Environmental Engineering Research Group, Faculty of Engineering and Business Turku University of Applied Sciences Turku Finland

## Abstract

Climate change poses significant consequences for temperate bat species, potentially altering their distribution ranges and generating novel interactions among species sharing similar ecological niches. Recent observations suggest distribution range expansion in the Palearctic aerial hawking bat, *Pipistrellus nathusii* , prompting an investigation into its interaction with *Eptesicus nilssonii* , a northern Palearctic species overlapping with the previous in many ecological characteristics. This study examines the spatiotemporal variations between the two boreal bat species to form an evidence‐based background onto which future research on, e.g., resource competition, can be built. A comprehensive community science project engaged over 470 participants from 45 high schools to collect acoustic data on bat echolocation calls across Finland, in northern Europe, during the summers of 2019–2020. Our modelling approach reveals distinct spatiotemporal patterns for each species. In early summer, *E. nilssonii* activity is concentrated in the southern region, whereas by late summer, observations are distributed across our study area, though predominantly in the south. This pattern suggests that *E. nilssonii* could exhibit post‐breeding vagrant behaviour, an observation only recently evidenced in bats. Conversely, the activity of *P. nathusii* remains notably low throughout the season, with observations concentrated along the south coast during both early and late seasons, making it challenging to fully model its distribution. Despite initial expectations of overlap given their similar foraging behaviour and habitat preferences, the limited activity and coastal association of *P. nathusii* suggest low competitive interaction with *E. nilssonii* . These findings contribute to our understanding of bat spatial ecology amid changing environmental conditions, emphasising the necessity for ongoing monitoring to ascertain the long‐term implications of shifting species distributions.

## Graphical abstract

Boreal bats are faced with very short summers during which to breed and gather fat reserves for the impending winter. Here, we show that boreal bats can move significant distances within a season to facilitate these behaviours.

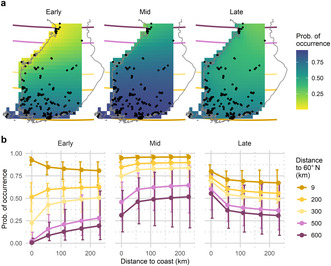

## 1 Introduction

Species distribution ranges are shifting dramatically due to climate change, particularly accentuated in the Northern Hemisphere, where these shifts have been documented across a variety of taxa, including plants (Kelly and Goulden 2008), fungi (Yan et al. 2017) and vertebrates (Lehikoinen and Virkkala 2016; Festa et al. 2023). However, these range shifts are not uniform in space and time. For instance, generalist species typically respond faster than specialist species with more restricted ecological niches (Brommer, Lehikoinen, and Valkama 2012), and even species sharing similar ecological traits may vary in their responses (Smeraldo et al. 2021), which can lead to the formation of novel interactions between species with differing responses to change. Therefore, range shifts have the potential to culminate in a competitive situation between species in similar ecological niches (Alexander, Diez, and Levine 2015). However, interspecies competition due to overlapping distribution ranges is rare, even in situations involving climate change–mediated range shifts. This is because other factors, such as spatiotemporal variation in habitat and landscape use between species, can effectively eliminate potential direct niche overlap (Gilman et al. 2010).

Among mammals, bats have a greater potential to respond to climate change through range shifts due to their ability to fly. Indeed, the distribution ranges of bats in Europe, for example, have been predicted to shift dramatically over the century (Rebelo, Tarroso, and Jones 2010). However, some species may not be able to respond effectively to climate change and could be experiencing population declines, whereas others are predicted to respond with significant increases in distribution range size (Festa et al. 2023). The Palearctic migratory species, *Pipistrellus nathusii* , is an example of the latter, exhibiting increased temporal presence at northerly latitudes (Kotila et al. 2023) and even evidence of hibernation in locations previously considered as summer breeding grounds (Blomberg et al. 2021). Alternatively, the northern Palearctic bat species, *Eptesicus nilssonii* , has shown a decrease in population size at the southern edge of its distribution range (Rydell et al. 2020) in southern Sweden. A reason for the decline has been attributed to increased competition with *Pipistrellus pygmaeus* , closely related to *P. nathusii* , and similarly rapidly increasing its range to the north.

A recent study revealed a significant overlap in distribution ranges of two aerial hawking bat species across Finland, situated to the northeast of Sweden (Kotila et al. 2023). Considering the potential decline in the population size of *E. nilssonii* in Sweden due to competition with *P. pygmaeus* , it is important to investigate whether increased competition with *P. nathusii* could similarly result in future declines in Finland. Despite similarities in traits such as wing loading (Froidevaux et al. 2023), diet (Krüger et al. 2014; Vesterinen et al. 2018), foraging habitat selection (Kalda, Kalda, and Liira 2015) and roost site selection (Hagner‐Wahlsten and Kyheröinen 2008; Suominen et al. 2023), the species differ greatly in their annual movement patterns, with *P. nathusii* being a long‐distance migrant (Vasenkov et al. 2022) and *E. nilssonii* associated with short‐ or medium‐distance annual movements (Suominen et al. 2020, 202). This can have marked effects on their phenology and spatiotemporal patterns, further influencing the likelihood of potential interactions between the species. Furthermore, whereas *E. nilssonii* is present across a variety of habitats (Vasko et al. 2020; Lundberg et al. 2021; Suominen et al. 2023), recent studies have suggested an affinity for the coastline in *P. nathusii* (Ijäs et al. 2017; Blomberg et al. 2021; Gaultier et al. 2023); a preference that has been observed in the species across its distribution range (Flaquer et al. 2009; Lundy, Montgomery, and Russ 2010; Bach et al. 2022). Whether this reflects a true affinity to the coastline, caused by bats aggregating there for seasonal migration (Ijäs et al. 2017), or if it is due to species expanding ranges across the Baltic Sea into Finland first encountering the coastline before moving inland towards higher latitudes, remains unknown due to a lack of spatiotemporal resolution in the data (Kotila et al. 2023).

Seasonal movements have also been suggested to occur in *E. nilssonii* , adding to the complexity of the spatiotemporal patterns in the species. For *E. nilssonii* , these movements appear to take place in late summer, after the nursery colonies disperse (Vasko et al. 2020; Kotila et al. 2023), during the pre‐hibernation fattening period (Fjelldal et al. 2024). The vagrant seasonal movements are presumed to have a northbound direction based on nationwide long‐term monitoring data (Kotila et al. 2023); however, the orientation of the monitoring setup utilised in the study only allows examining changes in bat activity along the north–south axis. Nevertheless, evidence also exists of late‐summer dispersal towards the Baltic Sea coastline in Finland (Rydell et al. 2014). Given the apparent complexity of spatiotemporal dynamics in both *E. nilssonii* and *P. nathusii* , including movement related to vagrant behaviour and migration, a study on overlap between the species requires a suitable monitoring network including both coastal and inland monitoring sites across both the latitudinal and longitudinal extents of Finland.

With the dynamics of bat distribution and behaviour becoming more complex with climate change (Festa et al. 2023), innovative approaches are needed to effectively monitor these changes. The use of community science (Ellwood et al. 2023), in which researchers are assisted by non‐scientists, has become increasingly useful in scientific research, especially with the recent achievements in technology (Barlow et al. 2015; Kosmala et al. 2016). Examples of large‐scale community science projects include, e.g., eBirds (https://ebird.org/home) and roostwatch (https://batwatch.ca/sp_canada). The introduction of low‐cost passive bat recorders provides opportunities to collect acoustic data across a large geographic range with the help of the community (Lundberg et al. 2021), providing fine‐scale resolution data when needed. In addition, the development of software that automatically identifies bat species based on their echolocation calls has made it possible to analyse a high volume of acoustic data (Rydell et al. 2017).

Here, we model the spatiotemporal occurrence of *P. nathusii* and *E. nilssonii* using acoustic data collected through a community science approach across Finland to produce baseline data for further assessment of the consequences of the rapid range expansion of *P. nathusii* on the population viability of *E. nilssonii* . We use latitude and distance to the coastline to model bat occurrence at sampling sites early, mid‐ and late summer to tease apart differences in phenology and spatial occurrence. Based on results from previous studies on the species (Ijäs et al. 2017; Vasko et al. 2020; Kotila et al. 2023; Suominen et al. 2023), we predict *P. nathusii* to show an affinity to the coastline that is not influenced by latitude, whereas *E. nilssonii* has a uniform distribution across the study area, with latitudinal variability in occurrence between the summer sub‐seasons due to vagrant behaviour.

## 2 Materials and Methods

### 2.1 Data Collection

From June till October in 2019 and 2020, we implemented a community science project aimed at Finnish high school students, in which students and their teachers (hereafter community scientists) helped researchers collect acoustic data. We recruited community scientists for this project by publishing an article about the project in Natura magazine (published by the association of biology and geography teachers, BMOL) and by contacting the schools directly. In 2019, 39 schools participated in the data collection and 18 in 2020. In addition, an independent individual not associated with schools participated in data collection. In total, about 470 community scientists and five researchers participated in acoustic data collection, which was conducted by using passive acoustic recorders (AudioMoths, https://www.openacousticdevices.info/audiomoth). AudioMoths are ideal for large‐scale community science projects due to their small size, low cost and ease of deliverability via mail.

We provided participants with all the equipment needed and detailed guidelines for data collection. The guidelines included information on the types of habitats where bats occur (e.g., proximity to water bodies, courtyards and parks and sparsely wooded areas were mentioned as possible bat habitats). We instructed community scientists on the places to avoid, such as areas with strong background noise (e.g., rapids), or anthropogenic noise (e.g., industrial area) or areas with power lines, or hard reflective surfaces (e.g., brick walls or asphalt areas) nearby. We also instructed them to avoid areas where crickets are present, as their frequency masks the echolocation sounds of bats (for details on the protocol, see Lundberg et al. (2021), Appendix S1). These all affect the quality of the sound collected and hence the detection of bats. Our guidelines also included instructions on how to use the AudioMoth device and how to deal with any problems that may arise during the data collection. If an AudioMoth device was damaged or lost during the data collection, we provided a replacement device to continue the data collection from the next time point onwards.

**FIGURE 1:**
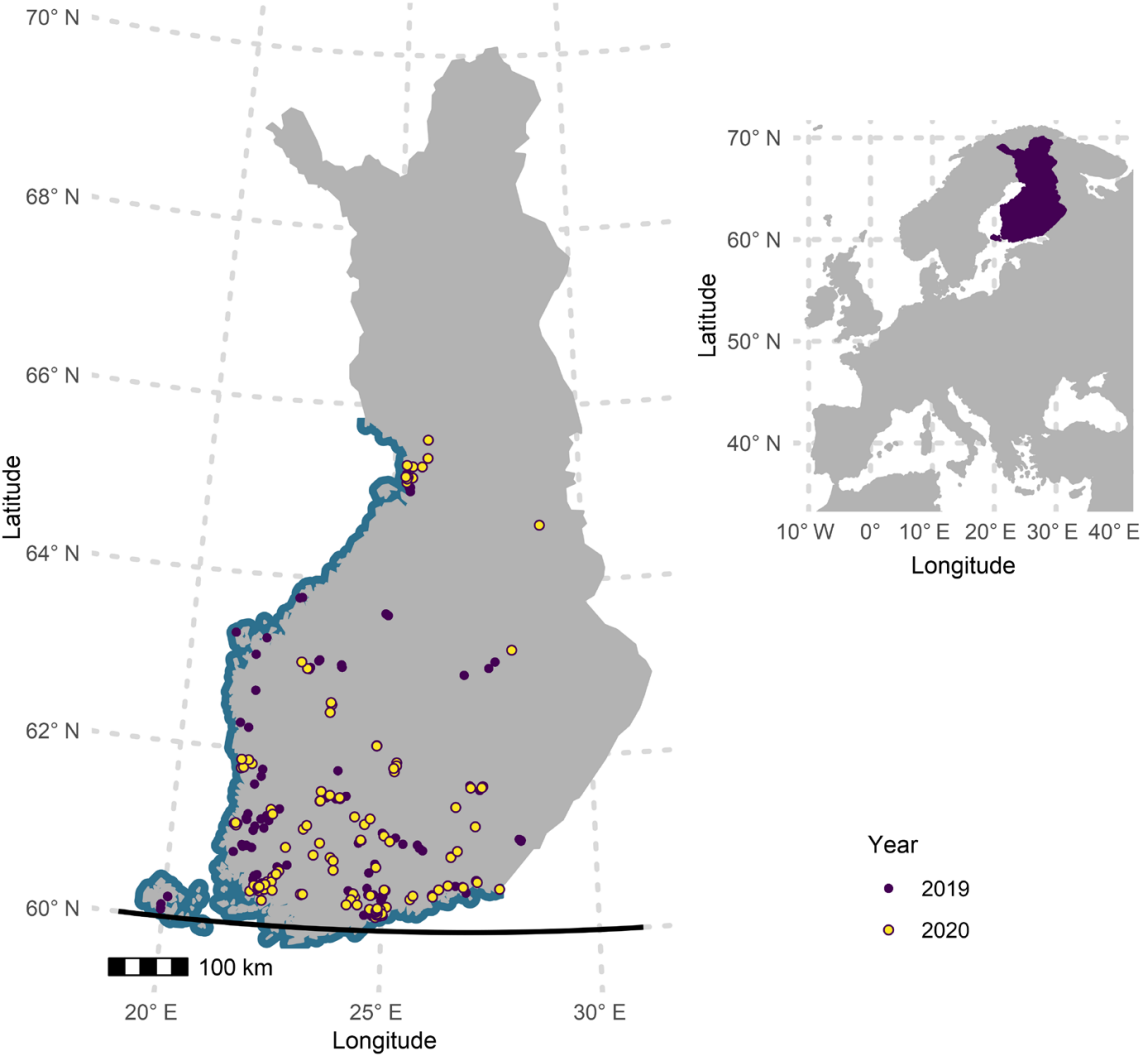
The location of the observation sites on a map of Finland in the summers of 2019 and 2020. The Baltic coast is highlighted with blue, and 60° N latitude is notated with a solid black line.

**FIGURE 2:**
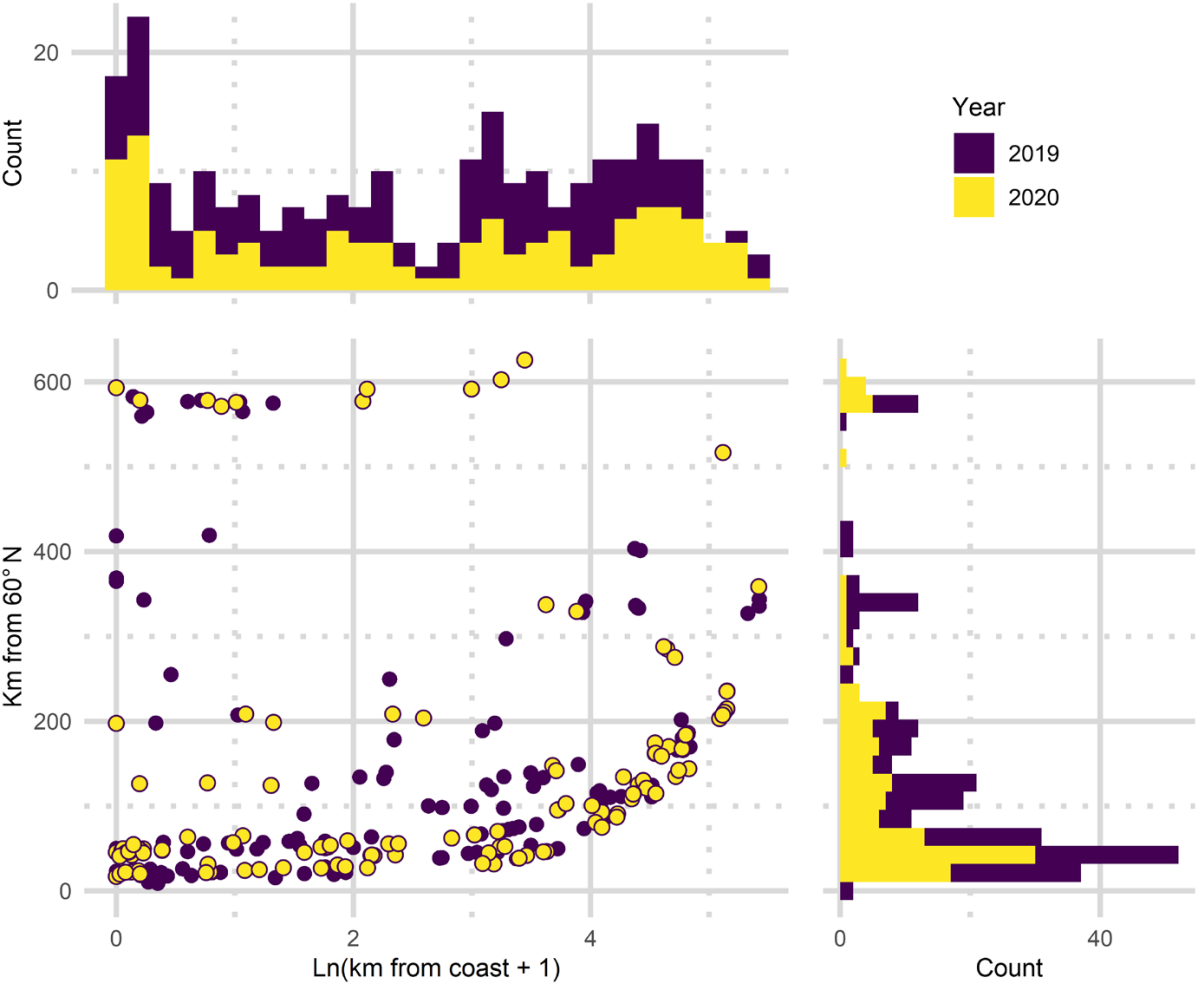
The observation sites in the summers of 2019 and 2020 in relation to the predictor variables used in the modelling: natural logarithm of distance to the Baltic coast (*x*‐axis) and distance to the 60° N latitude (*y*‐axis).

The data were collected from Southern to Central Finland covering the latitudes 60°–65° N and from the Åland Islands (latitude 60° N). The longitude for the westernmost observation site was 19° E, and for the easternmost site, it was 28° E. Devices were also scattered from the Baltic Sea coast to inland. The network of monitoring sites was the most dense at the southernmost latitudes and decreased northwards with notable aggregation around larger cities (Figures 1 and 2). Average distances (and standard deviations) between the sites were 214 km (135 km) in 2019 and 204 km (153 km) in 2020.

We used 140 and 127 AudioMoths for data collection in 2019 and 2020, respectively (Figure 1). All the participants registered the location of each observation site in the database maintained by the Finnish Biodiversity Information Facility (FinBIF, https://laji.fi/en), from which we obtained the coordinates of each study site. Altogether, each AudioMoth recorded up to 324 WAV‐files during the whole data collection period. Devices may have malfunctioned due to getting wet despite weather protection or the recording failed, resulting in missing data from a night or a recording period, which was accounted for in downstream analyses. In most cases, however, devices worked through the period (Appendix S1: Table S1). We pre‐programmed the devices with the entire data collection schedule to ensure synchronised collection across all locations. The recorders were in operation for a two‐night recording period every second week throughout the summer and were pre‐programmed to record for 10 min every half hour starting from 9.30 PM throughout the night totalling 18 files per night. The starting dates of the recording periods were 31st May, 14th June and 28th June (early season = recording periods 1–3), 12th July, 26th July and 9th August (mid‐season, recording periods 4–6) and 23rd August, 6th September and 20th September (late season, recording periods 7–9). The data collection dates were the same in both years.

### 2.2 Analyses of Acoustic Data

We used Kaleidoscope Pro (version 5.4.8) by Wildlife Acoustics Inc. (https://www.wildlifeacoustics.com/) and Bat Auto‐ID for species identification. We used noise filtering and split the recordings into 15‐s files. When running the analysis, the software compared echolocation calls from the recordings to inbuilt libraries of bat calls and classified the species based on reference calls. Finally, we manually checked the outputs from Kaleidoscope analysis and confirmed or corrected the result of the auto‐identification. For *E. nilssonii,* we checked all observations with a matching value below 2, while for *P. nathusii* , we examined all recorded observations. In addition, we checked all observations classified as NO‐ID (please see details of the protocol in Lundberg et al. (2021)).

### 2.3 Statistical Analyses

We used a generalised linear mixed model to describe the spatiotemporal patterns regarding latitude (i.e., distance from 60° N) and proximity to coast and compare their relative strength as predictors. Additionally, we included two categorical fixed variables: year (2019 or 2020) and sub‐season (early, mid‐ and late seasons). The early season would consist of recording periods 1–3, the mid‐season of periods 4–6 and the late season of periods 7–9. Separate models were fitted for *E. nilssonii* and *P. nathusii* . We included the observation site as the random intercept in the model because the unique surroundings of a location affect the detectability of bats. Additionally, a Matérn random intercept was added to account for spatial correlation between the observation sites. The modelling was performed in R version 4.2.0 (R Core Team 2022) using the package ‘spaMM’ version 4.1.20 (Rousset & Ferdy 2014).

We also fitted a series of simplified versions (null models) of the full model to justify including all fixed variables. The null models were 1. intercept only, 2. no interaction, 3. no sub‐season, 4. no year, 5. no proximity to coast and 6. no latitude. The null models and the full model were compared in terms of conditional AIC (spaMM package in R). For *E. nilssonii* , the full model had the lowest (i.e., best) cAIC and, thus, all predictors were retained. For *P. nathusii* , however, the 4th null model had the lowest cAIC and we therefore excluded the year‐predictor from the model formula (Table 3).

Proximity to the coast was calculated as the natural logarithm–transformed Euclidean distance to Baltic coast in kilometres. A logarithmic transformation was used to better reflect the previously observed effect, in which activity rapidly declines with growing distance from the coast (Ijäs et al. 2017). Prior to transformation, 1 km was added to each measurement to avoid negative values. To base all spatial data on the same projected coordinate reference system, Euclidean distance to the 60° N latitude was used in the modelling instead of geodetic latitude. All GIS operations were performed in R using the package ‘sf’ version 1.0‐7 and ETRS‐TM35FIN as the coordinate reference system. Tools in the package ‘tidyverse’ version 1.3.1 (Wickham et al. 2019) were used for data processing and visualisation throughout the script.

Bat activity was modelled as a Bernoulli trial with a logit link function, in which success was defined as 1 or more bat observations per period. We attempted the modelling of bat activity also as natural log‐transformed activity minutes per recording period (ln(n.min + 1)), but this approach resulted in poor fit judged from residual diagnostics from the package ‘DHARMa’ version 0.4.5. (Hartig and Lohse 2022). In the approach applied here, the activity values estimated by the model are probabilities of at least 1 bat being recorded at an observation site during a recording period, which consists of a 6‐h monitoring period.

Please see https://github.com/mhkoti/bats_by_the_coast for the complete R‐script for fitting and diagnosing the model and visualising the model estimates. We consider the data to fit the modelled predictors when the 95% confidence intervals for the fixed effects (ln‐distance to the coast and distance to the 60° N latitude) do not include zero. We used the function ‘confint.HLfit’ in ‘spaMM’ to calculate these intervals for all fixed effects.

## 3 Results

**FIGURE 3:**
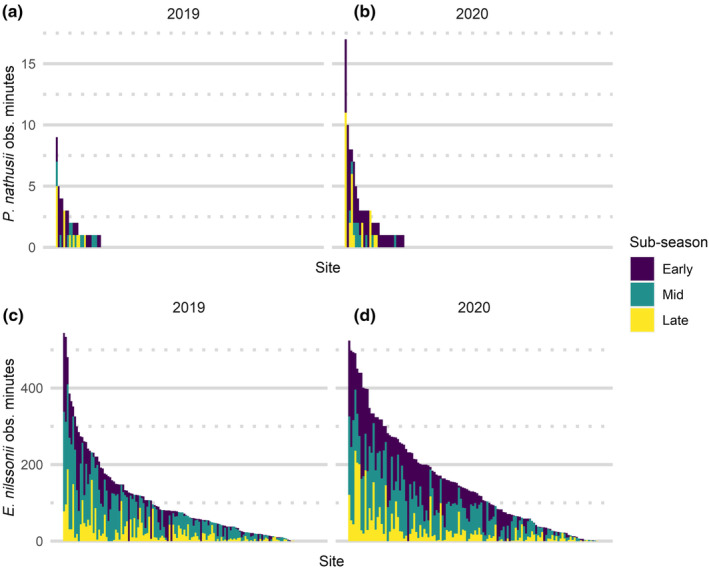
*Pipistrellus nathusii* observation minutes per site in (a) 2019 and (b) 2020 and *Eptesicus nilssonii* observation minutes per site in (c) 2019 and (d) 2020.

During a total of 11,818 monitoring hours, we recorded 32,381 min of *E. nilssonii* calls and 150 min of *P. nathusii* calls. On average, *E. nilssonii* was recorded 2.82 times per hour and *P. nathusii* was recorded 0.013 times per hour (Table 1). There are marked differences in per‐site bat activity, with a relatively few sites with high activity (Figure 3).

**TABLE 1:** Monitoring effort and observation minutes per focal species and period.

Period	Rec. hours		*Pipistrellus nathusii*				*Eptesicus nilssonii*			
			Obs. min		Obs. min/h		Obs. min		Obs. min/h	
	2019	2020	2019	2020	2019	2020	2019	2020	2019	2020
Early season	**2067**	**2034**	**13**	**350**	**0.006**	**0.170**	**3749**	**7591**	**1797**	**3740**
1	649	620	2	73	0.003	0.118	882	2372	1360	3830
2	732	699	8	132	0.011	0.189	1554	2691	2120	3850
3	686	715	3	145	0.004	0.203	1313	2528	1910	3540
Mid‐season	**1920**	**2113**	**13**	**99**	**0.007**	**0.047**	**7225**	**8316**	**3770**	**3923**
4	643	694	1	24	0.002	0.035	1651	1959	2570	2820
5	629	708	7	52	0.011	0.073	2861	3147	4550	4440
6	648	711	5	23	0.008	0.032	2713	3210	4190	4510
Late season	**1752**	**1932**	**30**	**96**	**0.017**	**0.049**	**3082**	**4051**	**1673**	**2017**
7	621	690	9	45	0.015	0.065	1899	2683	3060	3890
8	609	644	17	35	0.028	0.054	1136	1056	1870	1640
9	522	598	4	16	0.008	0.027	47	312	0.090	0.521
All seasons	**5739**	**6079**	**56**	**545**	**0.010**	**0.089**	**14,056**	**19,958**	**2413**	**3227**

*Note:* Numbers on the bolded lines are sums of the following lines, except for the ‘all‐seasons’ row, which is a summary of all other lines.

**FIGURE 4:**
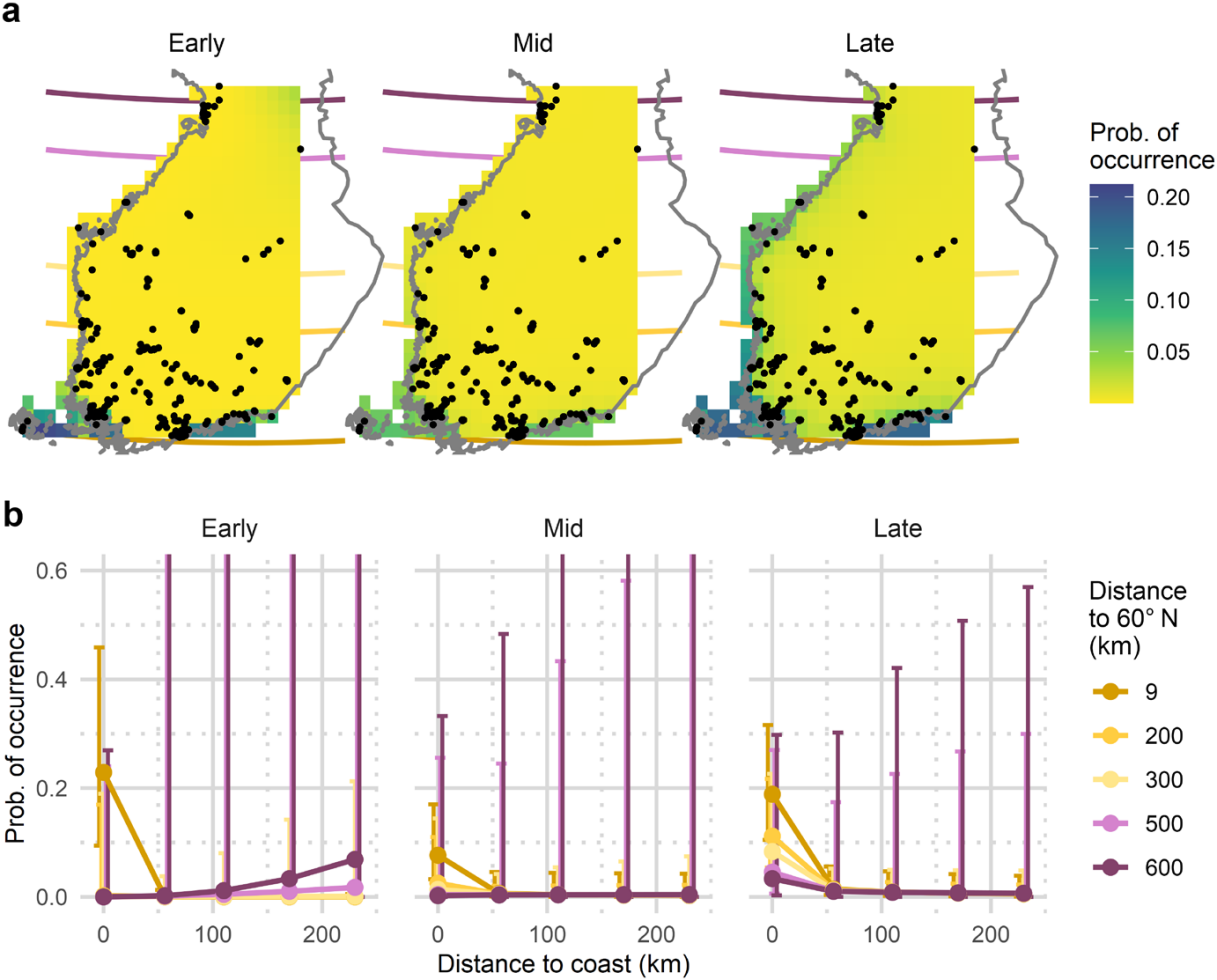
Model predictions for probability of occurrence of *Pipistrellus nathusii* mapped on a grid spanning the study area (a) and the same predictions presented as a line graph with 95% confidence intervals (b). The grid cells are 25 × 25 km, and their distance to the coast and latitude 60° N was measured from the centroid. The probability of occurrence in 2019 for each cell was calculated using the fixed effects. Estimates and 95% confidence intervals in the line graphs were calculated using the function ‘Effects’ in the R‐package ‘effects’ version 4.2‐2.

The modelling suggests that the occurrence of *P. nathusii* is explained by latitude, proximity to the coast, the sub‐season and their interactions (Table 2): According to the model, *P. nathusii* occurs exclusively on the southern coast through early and mid‐seasons (Figure 4a). In the late season, the predicted occurrence spreads along the entire Finnish Baltic coastline; however, the weight remains on the southern coast with about two times the probability of occurrence than 200 km higher in the north (Figure 4a). Even then, *P. nathusii* does not appear inland. The data also suggest a significant drop in occurrence during the mid‐season (Figure 4b and Table 2) as the probability of occurrence in the south is roughly half of what was estimated during early and late sub‐seasons.

**TABLE 2:** Estimates, *T*‐values and 95% confidence intervals for the fixed effects in the models.

Parameter	*Pipistrellus nathusii*					*Eptesicus nilssonii*				
	Estimate	*T*‐value	95% CI			Estimate	*T*‐value	95% CI		
			Lower	Upper				Lower	Upper	
Intercept	–0.984	−1.563	−2.162	0.304		2.394	6.968	1.735	3.104	*
Year 2020	—	—	—	—		0.464	2.660	0.116	0.820	*
Mid	–1.452	–1.997	–2.919	−0.162	*	0.325	0.723	−0.535	1.204	
Late	−0.446	−0.678	−1.800	0.728		−1.233	−3.296	−1.987	−0.505	*
North	−0.026	−1.846	−0.257	−0.007	*	−0.013	−5.781	−0.018	−0.009	*
ln(coast + 1)	−1.873	−3.141	−3.082	−0.930	*	−0.212	−1.714	−0.459	0.032	
Mid × north	0.020	1.347	−0.002	0.052		0.007	2.753	0.002	0.012	*
Late × north	0.022	1.606	0.003	0.055	*	0.011	4.632	0.007	0.016	*
Mid × ln(coast + 1)	1.226	1.863	0.178	2.500	*	0.262	1.561	−0.065	0.590	
Late × ln(coast + 1)	1.179	1.903	0.196	2.409	*	0.090	0.647	−0.187	0.370	
North × ln(coast + 1)	0.007	1.918	−0.002	0.014		0.001	2.204	0.000	0.003	*
Mid × north × ln(coast + 1)	−0.006	−1.417	−0.014	0.004		−0.001	−1.683	−0.003	0.000	
Late × north × ln(coast + 1)	−0.007	−1.679	−0.014	0.003		−0.002	−2.188	−0.003	0.000	*

*Note:* 95% confidence intervals for independent variables were calculated with the function ‘confint.HLfit’ in the package ‘spaMM’ version 4.1.20 (Rousset & Ferdy 2014) using R version 4.2.0 (R Core Team 2022). Asterisk denotes significance (i. e. confidence interval do not include zero).

While the highest‐predicted *P. nathusii* occurrence remains at the southern limit of the study area during the whole season (Figure 4), the proximity to coast is a much more important predictor of these two. In fact, including the latitude as a fixed effect barely improves the model fit with these data (Table 3). However, it has an impact as a modifier for proximity to coast. Moreover, the geography of Finland latitude and proximity to coast are partially codependent because the most southern points are situated by the sea (Figure 2). Moreover, the model predicts some occurrence in the northeastern corner of the study area; however, this should be ignored as an artefact given the lack of data in the area.

**TABLE 3:** Compared models and their conditional Akaike information criteria scores. Best model for each species studied is bolded.

Model description	Conditional AIC		Model predictors
	*P. nathusii*	*E. nilssonii*	
Full model	581.51	**1814.06**	Year + sub‐season × north × ln(coast + 1) + *Matern + site*
Intercept only	619.06	1980.18	*Matern + site*
No interaction	583.60	1858.15	Year + sub‐season + north + ln(coast + 1) + *Matern + site*
No sub‐season	605.16	1969.66	Year + north × ln(coast + 1) + *Matern + site*
No year	**580.31**	1816.94	Sub‐season × north × ln(coast + 1) + *Matern + site*
No coast	586.06	1820.93	Year + sub‐season × north + *Matern + site*
No north	581.99	1869.57	Year + sub‐season × ln(coast + 1) + *Matern + site*

**FIGURE 5:**
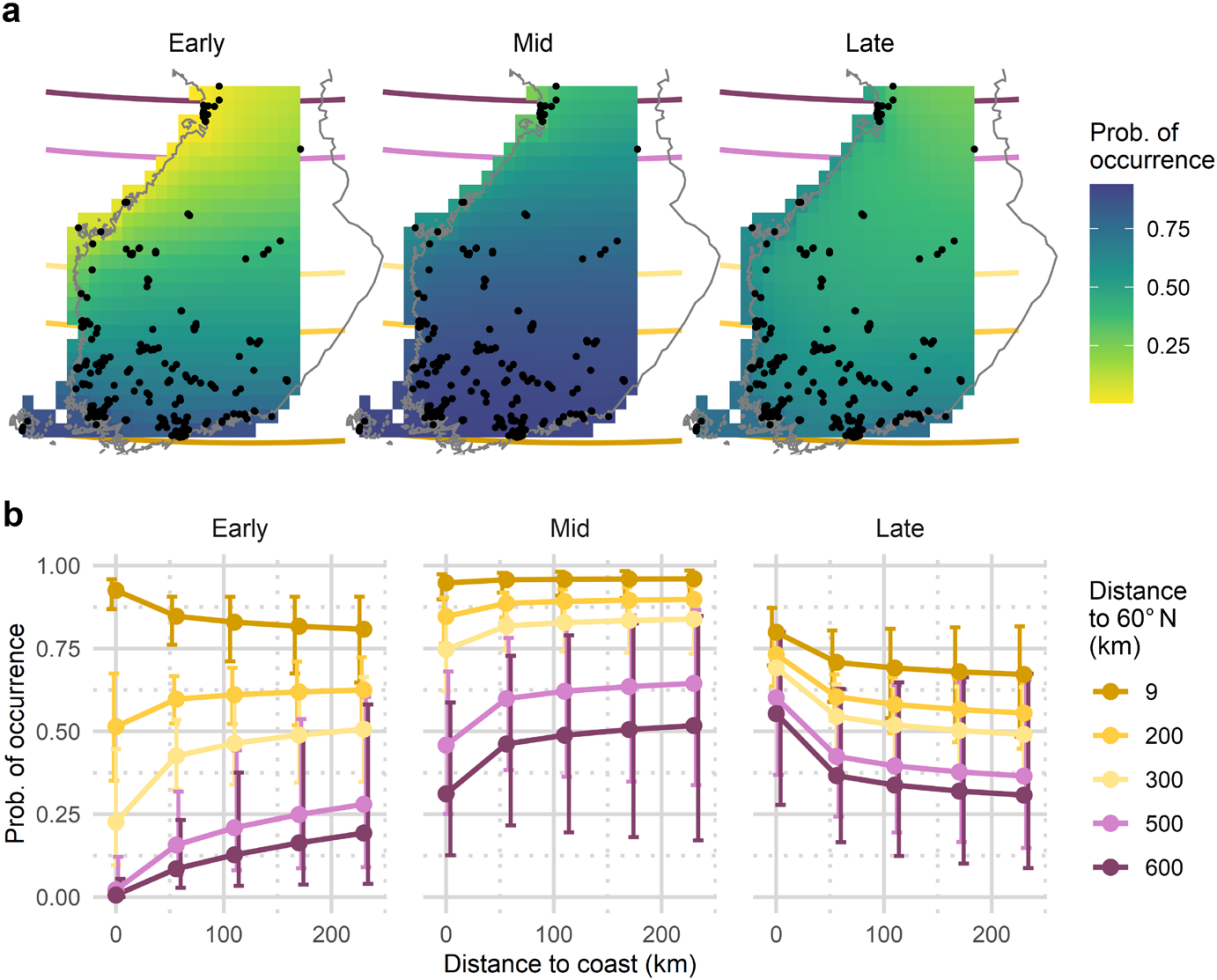
Model predictions for probability of occurrence of *Eptesicus nilssonii* mapped on a grid spanning the study area (a) and the same predictions presented as a line graph with 95% confidence intervals (b). The grid cells are 25 × 25 km, and their distance to the coast and the 60° N latitude was measured from the centroid. The probability of occurrence in 2019 for each cell was calculated using the fixed effects. Estimates and 95% confidence intervals in the line graphs were calculated using the function ‘Effects’ in the R‐package ‘effects’ version 4.2‐2.

According to the model, the predicted occurrence patterns of *E. nilssonii* are dominated by the interaction of latitude and sub‐season (Table 2). The predicted occurrence is negatively correlated with latitude regardless of the sub‐season (Figure 5). The difference in probability of occurrence between the north and south is most notable in the early season (from less than 0.25 to over 0.80), and this pattern gradually evens out towards the late season. However, the probability of occurrence in the north remains half of what is predicted in the south even in the late sub‐season. The southern part of the study area has the highest predicted occurrence for *E. nilssonii* during all sub‐seasons, but latitudinal differences are the smallest in the late season (Figure 5). In the south, *E. nilssonii* occurrence peaks in south during early and mid‐seasons. Contrastingly, the occurrence peak appears during mid‐ and late seasons in the north (Figure 5).

Proximity to coast has relatively small impact on the predicted occurrence of *E. nilssonii* (Figure 5). In the early season, occurrences by the coast are higher in the south but lower in the north. However, the monitoring sites are slightly biased towards the coastline both in the very north and very south (Figure 2), causing a lot of potential error in the inland estimates (Figure 5b). In the late season, all coastal areas have a higher‐predicted occurrence (Figure 5), but again, potential for error is high in comparison to the effect size (Figure 5b).

## 4 Discussion

Our extensive data collection indicates that *E. nilssonii* is the dominant species on the landscape at latitudes 60°–65°, while *P. nathusii* is less frequently detected by acoustic monitoring in our study area across Finland. The results further reveal distinct spatiotemporal occurrence patterns between the two species. As per our predictions, *Pipistrellus nathusii* is primarily found in the southern parts of the country in the vicinity of the Baltic Sea coastline in early and late seasons, with regular occurrences at higher latitudes on the coastline in the late season. The species is seldom observed during the mid‐season across the study range. In contrast, *E. nilssonii* shows a faint affinity to the coastline in the late season, but its occurrence exhibits a greater latitudinal variation across the sub‐seasons. Notably, *E. nilssonii* is heavily biased to the southern latitudes of our study area during the early season and levels off across the latitudes over mid‐ and late seasons.

Although seasonal migration has received a lot of attention in Europe only (Hutterer et al. 2005; Ahlen, Baagoe, and Bach 2009; Rydell et al. 2014; Voigt et al. 2015), the within‐season temporal movements of European bats are still poorly documented and understood. However, some acoustic studies suggest that bats translocate within their active season, with bats being abundant in given areas at different time points during the season (Hull and Cawthen 2013; Vasko et al. 2020; Kotila et al. 2023). The observed latitudinal pattern in *E. nilssonii* evidenced in our study may be best explained by vagrant behaviour (Tapia and Zuberogoitia 2018), observed by Rydell (Rydell 1993) as an increase in the solitary foraging behaviour. For instance, most of the known *E. nilssonii* breeding colonies in Finland are situated in the southern parts of the country (Suominen et al. 2023), which is consistent with the high occurrence recorded in our early‐season data. The southerly emphasis may be influenced by higher nighttime temperatures associated with prey availability and a larger proportion of dark hours during the night compared to more northern latitudes. However, *E. nilssonii* pups become volant in a matter of weeks (Fjelldal and van der Kooij 2024) and the breaking up of nursery colonies coincides with the rapid lengthening of nights and increasing temperatures at northerly latitudes, providing vagrant bats an abundance of arthropods (Mjaaseth et al. 2005).

Our data suggest that despite an increase in observations (Ijäs et al. 2017; Blomberg et al. 2020; Gaultier et al. 2023), the occurrence of *P. nathusii* in Finland is still two orders of magnitude lower than that of *E. nilssonii* . In addition, despite the species having been detected several times as far north as 69° in the late season (Kotila et al. 2023), our results here suggest that the activity in *P. nathusii* is positively affected by low latitude and proximity to the coast. Activity across the entire season was only recorded at sampling sites in the immediate proximity of the coast shown in our dataset, which is in accordance with our knowledge of breeding colonies for the species occurring only on the southern coast (Hagner‐Wahlsten and Kyheröinen 2008). Furthermore, a higher probability of overall occurrence and activity in the proximity of the coastline has been previously described in Finland (Ijäs et al. 2017; Gaultier et al. 2023) and elsewhere across the distribution range of the species (Flaquer et al. 2009; Lundy, Montgomery, and Russ 2010). The affinity to the coast shows temporal variation, with occurrence most probable in the late season. This most likely reflects the increase in population size due to breeding as well as the movement of migratory bats from inland sites to the coastline before migration (Desholm et al. 2014; Ijäs et al. 2017; Kruszynski et al. 2021). Finally, we cannot disclose the possibility that the close relationship of the species to the southern coastline is reflective of the recent settlement in Finland through range expansion with yet unrealised potential to disperse further north (Tidenberg, Liukko, and Stjernberg 2019).

Although only a faint tendency in *E. nilssonii* , both focal species show affinity to the coastline in the late season. In the case of *P. nathusii* , this is likely associated with migratory behaviour; however, the possibility of vagrant behaviour in both species in search of food, correlating with this trend, should be investigated in more detail. Although there are small lakes, ponds and rivers across Finland, and hence no lack of water, smaller water bodies cool down faster in the late summer (Lathrop et al. 2019), negatively impacting the emergence of aquatic insects. Preliminary evidence from the Kvarken archipelago in Finland suggests that bats are aggregating at the coast to feed on insects emerging from the brackish water Baltic Sea (Schneider and Fritzén 2020.) In fact, maximum insect abundance during the late season in the Kvarken archipelago positively correlates with the activity of *P. nathusii* , absent during other times of the year (Schneider and Fritzén 2020). Insect availability may also be responsible for other patterns in our data. For instance, the phenology of migratory insects can in part explain the evidenced of mid‐ and late‐season northward‐bound vagrant behaviours in *E. nilssonii* . Other factors may also be contributing to these observed patterns, such as differences in overwintering and breeding phenology (Reimer and Barclay 2024; Fjelldal and van der Kooij 2024) and smaller‐scale shifts in the habitat use across the active season (Vasko et al. 2020). Nevertheless, the movement of bats in relation to insect availability has been previously documented (McCracken et al. 2012; Gonsalves et al. 2013; Krauel, Westbrook, and McCracken 2015; Krauel et al. 2024); however, it has not been associated with a predictable vagrant behaviour as presented here and in Kotila et al. (2023). Similar seasonal vagrant behaviour has also been reported in falcons *Falco tinnunculus* and *Falco vespertinus* , in Fennoscandia (Galushin 1974). These nomads are often young individuals with movements linked to prey availability (Van Zyl, Jenkins, and Allan 1994). Little is known about insect migrations in Finland so far, and future research in the boreal zone could focus on gaining a better understanding of whether the late‐season movement of bats is associated with insect abundance.

The spatiotemporal patterns of the two focal species described in our study do not currently support the possibility of a similar competitive situation as suggested between *E. nilssonii* and *P. pygmaeus* in southern Sweden (Rydell et al. 2020), attributed to the population decline in *E. nilssonii* . We only observed a slight increase in overlapping occurrence at sampling sites in the late season and spatial segregation in early and mid‐seasons. Furthermore, the annual activity of *E. nilssonii* appears to be increasing in Finland (Kotila et al. 2023). Competition has often been regarded as the major force structuring communities and it also has major effects on the distribution of species (Abrams 1990); however, interspecific competition is difficult to prove in free‐living, highly mobile organisms such as bats (but see Arlettaz, Godat, and Meyer 2000; Roeleke, Johannsen, and Voigt 2018). There are several resources bats may compete for, such as food and foraging habitats, roosts, water and access to water bodies, as well as acoustic space (Salinas‐Ramos et al. 2020), but resources must be limited for them to lead to competition. It is difficult to estimate the amount of resources in relation to the number of bats. In northern Europe, bats have wider geographical range sizes as compared to southern Europe and fewer species (Meramo et al. 2023). The lack of competition has been proposed as a potential explanation for the broader species distributions observed at higher latitudes in northern Europe. Nevertheless, food scarcity, in the late season with declining insect abundances, may drive the use of space leading to exploitation competition (Roeleke, Johannsen, and Voigt 2018). It is noteworthy that with food or foraging areas being the cause of possible competition, exploitation competition may escalate in the future due to many different environmental changes, such as declines in insect populations or changes in land use. Thus, although our focal species may not yet be competing, they may soon face competition due to decreasing resources.

Regarding our sampling design, the population of Finland—and thus the schools participating in our study—is biased towards southern Finland, which is reflected in the geographic distribution of our devices. Therefore, the modelled patterns regarding the less sampled parts of the study area (Figures 4a and 5a) should be interpreted with some caution. Future projects could aim to cover areas of eastern and northern Finland that were only partially addressed by this study. Furthermore, the quality of collected data may vary when the public is involved in the research. However, our previous research suggests that detailed instructions, as used here, can facilitate the production of high‐quality results, comparable to that of professional researchers (Lundberg et al. 2021). Regardless, our community approach provided data with unparalleled resolution to reveal the patterns observed, thus warranting the further engagement of the community in data collection and science.

Knowledge on the spatial and temporal distribution of Finnish bats is beginning to increase (Tidenberg, Liukko, and Stjernberg 2019; Vasko et al. 2020; Kotila et al. 2023; Suominen et al. 2023), but due to changes in species composition and distribution ranges being accelerated by climate change, and an acceleration in energy production by wind and solar power (Brabant et al. 2019; Kruszynski et al. 2022; Gaultier et al. 2023), research providing both fine‐ and large‐resolution data on seasonal movements of bats is of immense importance. Here, we highlighted the importance of monitoring programmes and the importance of engaging the community to assist in providing high‐resolution data through technological advances in recording hardware to better understand spatiotemporal patterns of bats. The use of such methodology is strongly encouraged for monitoring shifts in bat diversity and distribution because of the rapid environmental change. The new information produced by our study has also practical applications. The occurrence of bats in the boreal zone varies spatially and temporally, not only for migratory species such as *P. nathusii* but also for species thus far considered to be sedentary, such as *E. nilssonii*. This means that environmental impact assessment guidelines should require monitoring to consider the entire active season of bats to better reflect this. This is of particular importance in planning of wind energy construction, for which curtailment plans are mostly based on data from migratory species (Gaultier et al. 2020).

## Author Contributions

**Piia Lundberg:** conceptualization (equal), data curation (leading), funding acquisition (supporting), investigation (equal), methodology (leading), project administration (supporting), supervision (equal), writing – original draft (equal), writing – review and editing (equal). **Miika Kotila:** conceptualization (equal), data curation (supporting), formal analysis (leading), validation (leading), visualization (leading), writing – original draft (equal), writing – review and editing (equal). **Katarina Meramo:** writing – original draft (equal), writing – review and editing (equal). **Kati M. Suominen:** methodology (supporting) writing – original draft (equal), writing – review and editing (equal). **Miina Suutari:** data curation (supporting), investigation (equal), methodology (equal), writing – review and editing (equal). **Tia‐Marie Pietikäinen:** investigation (equal), methodology (supporting), writing – review and editing (equal). **Ville Vasko:** data curation (supporting), investigation (equal), methodology (supporting), writing – review and editing (equal). **Thomas M. Lilley:** conceptualization (equal), funding acquisition (leading), investigation (equal), methodology (equal), project administration (leading), resources (leading), supervision (equal), writing – original draft (equal), writing – review and editing (equal).

## Conflicts of Interest

The authors declare no conflicts of interest.

## Supporting information


Appendix S1.

## Data Availability

Complete dataset and R code for replicating the modelling results are available at https://github.com/mhkoti/bats_by_the_coast.

## References

[ece370599-bib-0001] Abrams, P. A. 1990. “Ecological Vs Evolutionary Consequences of Competition.” Oikos 57: 147–151.

[ece370599-bib-0002] Ahlen, I. , H. J. Baagoe , and L. Bach . 2009. “Behavior of Scandinavian Bats During Migration and Foraging at Sea.” Journal of Mammalogy 90: 1318–1323.

[ece370599-bib-0003] Alexander, J. M. , J. M. Diez , and J. M. Levine . 2015. “Novel Competitors Shape Species' Responses to Climate Change.” Nature 525: 515–518. 26374998 10.1038/nature14952

[ece370599-bib-0004] Arlettaz, R. , S. Godat , and H. Meyer . 2000. “Competition for Food by Expanding Pipistrelle Bat Populations ( *Pipistrellus pipistrellus* ) Might Contribute to the Decline of Lesser Horseshoe Bats ( *Rhinolophus hipposideros* ).” Biological Conservation 93: 55–60.

[ece370599-bib-0005] Bach, P. , C. C. Voigt , M. Göttsche , et al. 2022. “Offshore and Coastline Migration of Radio‐Tagged Nathusius' Pipistrelles.” Conservation Science and Practice 4: e12783.

[ece370599-bib-0006] Barlow, K. E. , P. A. Briggs , K. A. Haysom , et al. 2015. “Citizen Science Reveals Trends in Bat Populations: The National Bat Monitoring Programme in Great Britain.” Biological Conservation 182: 14–26.

[ece370599-bib-0007] Blomberg, A. S. , V. Vasko , S. Salonen , G. Pētersons , and T. M. Lilley . 2020. “First Record of a Nathusius' Pipistrelle ( *Pipistrellus nathusii* ) Overwintering at a Latitude Above 60° N.” Mammalia 1. https://www.degruyter.com/view/journals/mamm/ahead‐of‐print/article‐10.1515‐mammalia‐2020‐0019/article‐10.1515‐mammalia‐2020‐0019.xml.

[ece370599-bib-0008] Blomberg, A. S. , V. Vasko , S. Salonen , G. Pētersons , and T. M. Lilley . 2021. “First Record of a Nathusius' Pipistrelle ( *Pipistrellus nathusii* ) Overwintering at a Latitude Above 60° N.” Mammalia 85: 74–78.

[ece370599-bib-0009] Brabant, R. , Y. Laurent , B. J. Poerink , and S. Degraer . 2019. “Activity and Behaviour of Nathusius' Pipistrelle *Pipistrellus nathusii* at Low and High Altitude in a North Sea Offshore Wind Farm.” Acta Chiropterologica 21: 341–348.

[ece370599-bib-0010] Brommer, J. E. , A. Lehikoinen , and J. Valkama . 2012. “The Breeding Ranges of Central European and Arctic Bird Species Move Poleward.” PLoS One 7: e43648. 23028465 10.1371/journal.pone.0043648 PMC3447813

[ece370599-bib-0011] Desholm, M. , R. Gill , T. Bøvith , and A. D. Fox . 2014. “Combining Spatial Modelling and Radar to Identify and Protect Avian Migratory Hot‐Spots.” Current Zoology 60: 680–691.

[ece370599-bib-0012] Ellwood, E. R. , G. B. Pauly , J. Ahn , et al. 2023. “Citizen Science Needs a Name Change.” Trends in Ecology & Evolution 38: 485–489. 37088666 10.1016/j.tree.2023.03.003

[ece370599-bib-0013] Festa, F. , L. Ancillotto , L. Santini , et al. 2023. “Bat Responses to Climate Change: A Systematic Review.” Biological Reviews 98: 19–33. 36054527 10.1111/brv.12893 PMC10087939

[ece370599-bib-0014] Fjelldal, M. A. , N. R. Fritzén , K. M. Suominen , and T. M. Lilley . 2024. “Supersize Me: Hypotheses on Torpor‐Assisted Prehibernation Fattening in a Boreal Bat.” Biology Letters 20: 20240291. 39288816 10.1098/rsbl.2024.0291 PMC11407865

[ece370599-bib-0015] Fjelldal, M. A. , and J. van der Kooij . 2024. “Individual Variation in Breeding Phenology and Postnatal Development in Northern Bats (*Eptesicus nilssonii*).” bioRxiv. https://www.biorxiv.org/content/10.1101/2024.05.22.595341v1. 10.1002/ece3.70324 PMC11499213 39450154

[ece370599-bib-0016] Flaquer, C. , X. Puig‐Montserrat , U. Goiti , F. Vidal , A. Curcó , and D. Russo . 2009. “Habitat Selection in Nathusius' Pipistrelle ( *Pipistrellus nathusii* ): The Importance of Wetlands.” Acta Chiropterologica 11: 149–155.

[ece370599-bib-0017] Froidevaux, J. S. P. , N. Toshkova , L. Barbaro , et al. 2023. “A Species‐Level Trait Dataset of Bats in Europe and Beyond.” Scientific Data 10: 253. 37137926 10.1038/s41597-023-02157-4 PMC10156679

[ece370599-bib-0018] Galushin, V. M. 1974. “Synchronous Fluctuations in Populations of Some Raptors and Their Prey.” Ibis 116: 127–134.

[ece370599-bib-0019] Gaultier, S. P. , A. S. Blomberg , A. Ijäs , et al. 2020. “Bats and Wind Farms: The Role and Importance of the Baltic Sea Countries in the European Context of Power Transition and Biodiversity Conservation.” Environmental Science & Technology 54: 10385–10398. 32830494 10.1021/acs.est.0c00070 PMC7497642

[ece370599-bib-0020] Gaultier, S. P. , T. M. Lilley , E. J. Vesterinen , and J. E. Brommer . 2023. “The Presence of Wind Turbines Repels Bats in Boreal Forests.” Landscape and Urban Planning 231: 104636.

[ece370599-bib-0021] Gilman, S. E. , M. C. Urban , J. Tewksbury , G. W. Gilchrist , and R. D. Holt . 2010. “A Framework for Community Interactions Under Climate Change.” Trends in Ecology & Evolution 25: 325–331. 20392517 10.1016/j.tree.2010.03.002

[ece370599-bib-0022] Gonsalves, L. , B. Law , C. Webb , and V. Monamy . 2013. “Foraging Ranges of Insectivorous Bats Shift Relative to Changes in Mosquito Abundance.” PLoS One 8: e64081. 23667699 10.1371/journal.pone.0064081 PMC3646781

[ece370599-bib-0023] Hagner‐Wahlsten, N. , and E.‐M. Kyheröinen . 2008. “First Observation of Breeding Nathusius' Pipistrelle ( *Pipistrellus nathusii* ) in Finland.” Memoranda Societatis pro Fauna et Flora Fennica 84: 36–40.

[ece370599-bib-0024] Hartig, F. , and L. Lohse . 2022. “DHARMa: Residual Diagnostics for Hierarchical (Multi‐Level/Mixed) Regression Models.” https://CRAN.R‐project.org/package=DHARMa.

[ece370599-bib-0025] Hull, C. L. , and L. Cawthen . 2013. “Bat Fatalities at Two Wind Farms in Tasmania, Australia: Bat Characteristics, and Spatial and Temporal Patterns.” New Zealand Journal of Zoology 40: 5–15.

[ece370599-bib-0026] Hutterer, R. , T. Ivanova , T. Meyer‐Cords , and L. Rodrigues . 2005. “Bat Migration in Europe.” Federal Agency for Nature Conservation.

[ece370599-bib-0027] Ijäs, A. , A. Kahilainen , V. V. Vasko , and T. M. Lilley . 2017. “Evidence of the Migratory Bat, *Pipistrellus nathusii* , Aggregating to the Coastlines in the Northern Baltic Sea.” Acta Chiropterologica 19: 127–139.

[ece370599-bib-0028] Kalda, O. , R. Kalda , and J. Liira . 2015. “Multi‐Scale Ecology of Insectivorous Bats in Agricultural Landscapes.” Agriculture, Ecosystems & Environment 199: 105–113.

[ece370599-bib-0029] Kelly, A. E. , and M. L. Goulden . 2008. “Rapid Shifts in Plant Distribution With Recent Climate Change.” Proceedings of the National Academy of Sciences 105: 11823–11826. 10.1073/pnas.0802891105 PMC2575286 18697941

[ece370599-bib-0030] Kosmala, M. , A. Wiggins , A. Swanson , and B. Simmons . 2016. “Assessing Data Quality in Citizen Science.” Frontiers in Ecology and the Environment 14: 551–560.

[ece370599-bib-0031] Kotila, M. , K. M. Suominen , V. V. Vasko , et al. 2023. “Large‐Scale Long‐Term Passive‐Acoustic Monitoring Reveals Spatio‐Temporal Activity Patterns of Boreal Bats.” Ecography 2023: e06617.

[ece370599-bib-0032] Krauel, J. J. , D. R. Reynolds , J. K. Westbrook , and G. F. McCracken . 2024. “Chapter 8 – Insect Migrations and the Ecology, Behavior, and Population Dynamics of Bats.” In A Natural History of Bat Foraging, edited by D. Russo and B. Fenton , 139–156. Academic Press. https://www.sciencedirect.com/science/article/pii/B978032391820600005X.

[ece370599-bib-0033] Krauel, J. J. , J. K. Westbrook , and G. F. McCracken . 2015. “Weather‐Driven Dynamics in a Dual‐Migrant System: Moths and Bats.” Journal of Animal Ecology 84: 604–614. 25492132 10.1111/1365-2656.12327

[ece370599-bib-0034] Krüger, F. , E. L. Clare , W. O. C. Symondson , O. Keišs , and G. Pētersons . 2014. “Diet of the Insectivorous Bat *Pipistrellus nathusii* During Autumn Migration and Summer Residence.” Molecular Ecology 23: 3672–3683. 24118366 10.1111/mec.12547

[ece370599-bib-0035] Kruszynski, C. , L. D. Bailey , L. Bach , et al. 2022. “High Vulnerability of Juvenile Nathusius' Pipistrelle Bats ( *Pipistrellus nathusii* ) at Wind Turbines.” Ecological Applications 32: e2513. 34877754 10.1002/eap.2513

[ece370599-bib-0036] Kruszynski, C. , L. D. Bailey , A. Courtiol , et al. 2021. “Identifying Migratory Pathways of Nathusius' Pipistrelles ( *Pipistrellus nathusii* ) Using Stable Hydrogen and Strontium Isotopes.” Rapid Communications in Mass Spectrometry 35: e9031. 33336436 10.1002/rcm.9031

[ece370599-bib-0037] Lathrop, R. C. , P. Kasprzak , M. Tarvainen , et al. 2019. “Seasonal Epilimnetic Temperature Patterns and Trends in a Suite of Lakes From Wisconsin (USA), Germany, and Finland.” Inland Waters 9: 471–488. 10.1080/20442041.2019.1637682.

[ece370599-bib-0038] Lehikoinen, A. , and R. Virkkala . 2016. “North by North‐West: Climate Change and Directions of Density Shifts in Birds.” Global Change Biology 22: 1121–1129. 26691578 10.1111/gcb.13150

[ece370599-bib-0039] Lundberg, P. , M. B. Meierhofer , V. Vasko , et al. 2021. “Next‐Generation Ultrasonic Recorders Facilitate Effective Bat Activity and Distribution Monitoring by Citizen Scientists.” Ecosphere 12: e03866.

[ece370599-bib-0040] Lundy, M. , I. Montgomery , and J. Russ . 2010. “Climate Change‐Linked Range Expansion of Nathusius' Pipistrelle Bat, *Pipistrellus nathusii* (Keyserling & Blasius, 1839).” Journal of Biogeography 37: 2232–2242.

[ece370599-bib-0041] McCracken, G. F. , J. K. Westbrook , V. A. Brown , M. Eldridge , P. Federico , and T. H. Kunz . 2012. “Bats Track and Exploit Changes in Insect Pest Populations.” PLoS One 7: e43839. 22952782 10.1371/journal.pone.0043839 PMC3432057

[ece370599-bib-0042] Meramo, K. , M. Kotila , S. Gaultier , et al. 2023. “Latitudinal Gradient and Species Traits Determine Bat Distributions Across Europe.” Preprints. https://www.authorea.com/users/594423/articles/628908‐latitudinal‐gradient‐and‐species‐traits‐determine‐bat‐distributions‐across‐europe?commit=90c4f18bbcfee847aa4943c788bc163f7822ef66.

[ece370599-bib-0044] Mjaaseth, R. R. , S. B. Hagen , N. G. Yoccoz , and R. A. Ims . 2005. “Phenology and Abundance in Relation to Climatic Variation in a Sub‐Arctic Insect Herbivore–Mountain Birch System.” Oecologia 145: 53–65. 16003503 10.1007/s00442-005-0089-1

[ece370599-bib-0045] R Core Team . 2022. “R: A Language and Environment for Statistical Computing.” R Foundation for Statistical Computing, Vienna, Austria. http://www.R‐project.org/.

[ece370599-bib-0046] Rebelo, H. , P. Tarroso , and G. Jones . 2010. “Predicted Impact of Climate Change on European Bats in Relation to Their Biogeographic Patterns.” Global Change Biology 16: 561–576.

[ece370599-bib-0047] Reimer, J. P. , and R. M. R. Barclay . 2024. “Seasonal Phenology of the Little Brown Bat ( *Myotis lucifugus* ) at 60° N.” Ecosphere 15: e4778.

[ece370599-bib-0048] Roeleke, M. , L. Johannsen , and C. C. Voigt . 2018. “How Bats Escape the Competitive Exclusion Principle—Seasonal Shift From Intraspecific to Interspecific Competition Drives Space Use in a bat Ensemble.” Frontiers in Ecology and Evolution 6. 10.3389/fevo.2018.00101.

[ece370599-bib-0067] Rousset, F. and Ferdy, J.‐B . 2014. “Testing environmental and genetic effects in the presence of spatial autocorrelation.” Ecography 37: 781–790.

[ece370599-bib-0049] Rydell, J. 1993. “Variation in Foraging Activity of an Aerial Insectivorous Bat During Reproduction.” Journal of Mammalogy 74: 503–509.

[ece370599-bib-0050] Rydell, J. , L. Bach , P. Bach , et al. 2014. “Phenology of Migratory Bat Activity Across the Baltic Sea and the South‐Eastern North Sea.” Acta Chiropterologica 16: 139–147.

[ece370599-bib-0051] Rydell, J. , M. Elfström , J. Eklöf , and S. Sánchez‐Navarro . 2020. “Dramatic Decline of Northern Bat *Eptesicus nilssonii* in Sweden Over 30 Years.” Royal Society Open Science 7: 191754. 32257332 10.1098/rsos.191754 PMC7062070

[ece370599-bib-0052] Rydell, J. , S. Nyman , J. Eklöf , G. Jones , and D. Russo . 2017. “Testing the Performances of Automated Identification of Bat Echolocation Calls: A Request for Prudence.” Ecological Indicators 78: 416–420.

[ece370599-bib-0053] Salinas‐Ramos, V. B. , L. Ancillotto , L. Bosso , V. Sánchez‐Cordero , and D. Russo . 2020. “Interspecific Competition in Bats: State of Knowledge and Research Challenges.” Mammal Review 50: 68–81.

[ece370599-bib-0054] Schneider, M. , and N. Fritzén . 2020. “Flador Och Deras Insektproduktion – Betydelsen för Lokala Och Migrerande Fladdermöss i Kvarken.”

[ece370599-bib-0055] Smeraldo, S. , L. Bosso , V. B. Salinas‐Ramos , et al. 2021. “Generalists Yet Different: Distributional Responses to Climate Change May Vary in Opportunistic Bat Species Sharing Similar Ecological Traits.” Mammal Review 51: 571–584.

[ece370599-bib-0056] Suominen, K. M. , M. Kotila , A. S. Blomberg , H. Pihlström , V. Ilyukha , and T. M. Lilley . 2020. “Northern Bat *Eptesicus nilssonii* (Keyserling and Blasius, 1839).” In Handbook of the Mammals of Europe, edited by K. Hackländer and F. E. Zachos , 1–27. Cham: Springer International Publishing. 10.1007/978-3-319-65038-8_45-1.

[ece370599-bib-0057] Suominen, K. M. , E. J. Vesterinen , I. Kivistö , et al. 2023. “Environmental Features Around Roost Sites Drive Species‐Specific Roost Preferences for Boreal Bats.” Global Ecology and Conservation 46: e02589.

[ece370599-bib-0058] Tapia, L. , and I. Zuberogoitia . 2018. “Breeding and Nesting Biology in Raptors.” In Birds of Prey: Biology and Conservation in the XXI Century, edited by J. H. Sarasola , J. M. Grande , and J. J. Negro , 63–94. Cham: Springer International Publishing. 10.1007/978-3-319-73745-4_3.

[ece370599-bib-0059] Tidenberg, E.‐M. , U.‐M. Liukko , and T. Stjernberg . 2019. “Atlas of Finnish Bats.” Annales Zoologici Fennici 56: 207–250.

[ece370599-bib-0060] Van Zyl, A. J. , A. R. Jenkins , and D. G. Allan . 1994. “Evidence for Seasonal Movements by Rock Kestrels Falco Tinnunculus and Lanner Falcons *F. biarmicus* in South Africa.” Ostrich 65: 111–121.

[ece370599-bib-0061] Vasenkov, D. , J.‐F. Desmet , I. Popov , and N. Sidorchuk . 2022. “Bats Can Migrate Farther Than It Was Previously Known: A New Longest Migration Record by Nathusius' Pipistrelle *Pipistrellus nathusii* (Chiroptera: Vespertilionidae).” Mammalia 86: 524–526.

[ece370599-bib-0062] Vasko, V. , A. S. Blomberg , E. J. Vesterinen , et al. 2020. “Within‐Season Changes in Habitat Use of Forest‐Dwelling Boreal Bats.” Ecology and Evolution 10: 4164–4174. 32489639 10.1002/ece3.6253 PMC7244798

[ece370599-bib-0063] Vesterinen, E. J. , A. I. E. Puisto , A. S. Blomberg , and T. M. Lilley . 2018. “Table for Five, Please: Dietary Partitioning in Boreal Bats.” Ecology and Evolution 8: 10914–10937. 30519417 10.1002/ece3.4559 PMC6262732

[ece370599-bib-0064] Voigt, C. C. , L. S. Lehnert , G. Petersons , F. Adorf , and L. Bach . 2015. “Wildlife and Renewable Energy: German Politics Cross Migratory Bats.” European Journal of Wildlife Research 61: 213–219.

[ece370599-bib-0065] Wickham, H. , M. Averick , J. Bryan , et al. 2019. “Welcome to the Tidyverse.” Journal of Open Source Software 4: 1686.

[ece370599-bib-0066] Yan, Y. , Y. Li , W.‐J. Wang , et al. 2017. “Range Shifts in Response to Climate Change of Ophiocordyceps Sinensis, a Fungus Endemic to the Tibetan Plateau.” Biological Conservation 206: 143–150.

