# Supplementary material for: Contrasting Seasonal Distribution Patterns of Two Boreal Aerial Hawking Bat Species in Finland
Source: Ecol Evol. 2025 Jan 16;15(1):e70599. doi: 10.1002/ece3.70599 (PMC11738648; doi:10.1002/ece3.70599)
Supplement: Supplementary file 1 — Appendix S1. [file ECE3-15-e70599-s001.docx]

**Appendix 1**

Contents

[Figure S1 A photo collage of instructions that were given to participants in the project’s Instagram account. 2](#_Toc179531433)

[Table S1 Number and percentage of the active devices per weekend and year 3](#_Toc179531434)


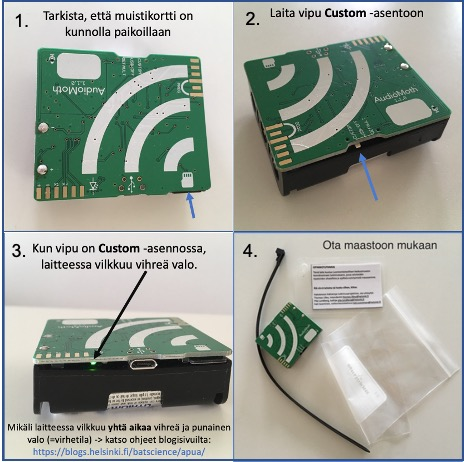


# Figure S1 A photo collage of instructions that were given to participants in the project’s Instagram account.

Table S1 Number and percentage of the active devices per weekend and year. Only the devices that worked flawlessly through given recording period are considered active and included in the numbers.

|  | N active devices | |  | % active devices | |
| --- | --- | --- | --- | --- | --- |
| Rec. period | 2019 | 2020 |  | 2019 | 2020 |
| 1 | 105 | 101 |  | 76 | 80 |
| 2 | 118 | 116 |  | 85 | 91 |
| 3 | 111 | 117 |  | 80 | 92 |
| 4 | 103 | 113 |  | 74 | 89 |
| 5 | 100 | 118 |  | 72 | 93 |
| 6 | 107 | 118 |  | 77 | 93 |
| 7 | 103 | 115 |  | 74 | 91 |
| 8 | 97 | 105 |  | 70 | 83 |
| 9 | 80 | 97 |  | 58 | 76 |
| all | 924 | 1000 |  | 81 | 80 |
